# 

**Published:** 1879-04-20

**Authors:** 


RECEIPTED.—1877—G. A. B. Hays. 1878—Drs. J. F. Pou, W. W. Coggin 6ms; M. B. Pollard; W. A. Dobbs; J. H. Payne; S. L. Singleterry; T. E. Morris; J. F. Davis; Jno. O. Brien; R. S. Hullsey; L. G. Lincecum; V. Lockheart, 6ms.; T J Parker; T. E. Collier; G. M. Rivers; R. D. Lucius. 1878-9—W. B. Maxwell; T. N. Skeen; W. R. Robison; W. T. Beall; C. M. Norwood; W. W. Farr; J. S. Green; C. W. Keller; T. J. Charlton; I N Gilmore; R. S. Powell. 1879—J. A. Holloway; C. P. Sanders; A. L. Hawkins; R. E. Hntchins; Wm. Whitehead, Lanier & McCabe; J. E. Carter, to July 1st.; J. W. Burt; P. S Anderson, W. Culpepper; J. C. McCoy; John J. Gage; J. F. Earnest, 6 ms.; M. T. Bell; D. J. Parsons; J. C. Bivings; J. T. and J. F. Alford; E. Wheeler; A. J. Kobb; J. M. Strong; Jos. Underwood; J. C. Boozer, P. Taylor; W. H. Stewart, 6 ms; Tho. H. Roberds; R. J. Tofbert; J. W. Thomas; F. J. Thorpe; T. C. Powell; T. E. Pennington, 6 ms. John Elsner; Dr. Brownlee; J. W. Harris; W. E. Brock; A. F. Fricks; J. T. Persons; Tho. B. Meacham; K. H. Davis; J. C. Wallace; S. N. Bruce; C. J. Burroughs; J. A. Fields; W. E. Saterfield; J. I. Grover; Louis Hadden; T. A. Cook; J. F. Davis; A. H. Sellers; S. Y. Carter; F. M. Bledsœ; E. H. McGehee; L. D. Webb; C. M. Gibson; Booth & Jarrett; E. R. Young; J. W. Price; S. A. Berry; W. W. Elrod; R. A. Sells; E. A. Anderson; J. L. Selman; H. Allison; M. J. Luster; R. A. Shimssock; M. B. Miller; J. K. Davidsons; F. M. Rushing; B. E. Clark; C. M. Bold s; H. C. Walkup; J. D Johnson; S. M. Cummings; J. D. Harwell; M. E. Demaret; E. H. Parham; Holderness & Robertson; D. S. Aswell; A. C. Simonton; S. H. Dilburn; C. Wall; J. G. Donald; J. W. Baker; J. A. Agnew; A. W. Agnew; W. F. Morrison; J. W. Ethridge; J. W. Bunt; T. E. Morris; S. V. Parker; T. C. Davis; M. L. Barron; C. T. Coleman; P. P. Terry; E. F. Sumerall. 1879-80—J. E. Trippe.

